# Advance Care Planning: A Story of Trust Within the Family

**DOI:** 10.1177/07334648231214905

**Published:** 2023-11-20

**Authors:** Lory A. Iunius, Sarah Vilpert, Clément Meier, Ralf J. Jox, Gian Domenico Borasio, Jürgen Maurer

**Affiliations:** 1 Faculty of Business and Economics (HEC), University of Lausanne, Switzerland; 2 Swiss Centre of Expertise in the Social Sciences (FORS), Lausanne, Switzerland; 3 Palliative and Supportive Care Service, Lausanne University Hospital and University of Lausanne, Switzerland; 4 Institute of Humanities in Medicine, Lausanne University Hospital and University of Lausanne, Switzerland

**Keywords:** trust, family, advance directives, population-based study, advance care planning

## Abstract

As the family usually plays a central role at the end of life, the quality of family relationships may influence how individuals approach advance care planning (ACP). Our study investigates the associations of trust in relatives with regard to end-of-life (EOL) issues—used as a proxy measure of family relationship quality—with individuals’ engagement in EOL discussions, advance directive (AD) awareness, approval and completion, and designation of a healthcare proxy. Using nationally representative data of adults aged 55 years and over from wave 6 (2015) of the Survey of Health, Ageing, and Retirement in Europe (SHARE) in Switzerland (*n* = 1911), we show that complete trust in relatives is related to higher engagement in ACP. Subject to patient consent, the family should, therefore, be included in the ACP process, as such practice could enhance patient-centered EOL care and quality of life at the end of life.

**What this paper adds**

• This manuscript provides new insight into understanding the importance of high-quality family ties in end-of-life care planning in the older adult population.
• Specifically, little is known about the quality of family relationships and its association with ACP aspects in Switzerland. This study helps to fill this gap.
• The findings of our study reveal that even in individualistic societies, the family maintains an essential place in end-of-life and death-related matters, especially when relationships are good.

**Applications of study findings**

• The study findings highlight the role of the family in the different stages of ACP. The family can serve as a source of motivation and support in this process.
• The entire population should be made aware of ACP as potential partners, children, brothers, or sisters who can encourage, accompany, and support their relatives in the ACP process. This draws on the invaluable ties of trust relatives may develop. Given the family’s place in the EOL process, professionals involved in EOL care and planning should systematically address the issue of including family members, or trusted people, in EOL discussions, decisions, and care planning with the patient.

## Introduction

At the end of life, individuals are often confronted with complex and unique challenges that may threaten their physical and emotional integrity. It is widely recognized that informal carers have an essential function during the highly sensitive and critical period that precedes death by accompanying the patients through their illness experience and providing vital care and emotional support (Goldberg, 2010; Grande et al., 2009). Informal caregivers are mainly family members (Costello, 2017), and it appears that family structure, dynamics, and involvement can strongly influence individuals’ experiences as death approaches (Peterson et al., 2019). For example, the ability to keep a person with a life-threatening illness at home and provide palliative care in this setting often depends on the family’s ability to organize itself to deliver all the necessary care that is not offered by professional caregivers (Broom & Kirby, 2013; Stajduhar, 2013). Furthermore, relatives who accompany their loved ones during illness often support them in making medical decisions and are sometimes asked to make substitute medical decisions for those who have lost decision-making capacity (Meeker & Jezewski, 2005; Trees et al., 2017). These examples illustrate that dying is, to a large part, a relational process (Broom & Kirby, 2013) in which family caregivers represent an important resource. Given the central role of the family in the end-of-life (EOL) context, it is important to understand how family members are involved in the advance care planning (ACP) process, which depends on more than just physician-patient relationships (van Eechoud et al., 2014).

According to the hierarchical compensatory model proposed by Cantor (1979), older adults tend to have a hierarchical preference for assistance and support from family members in a variety of situations (health, financial tasks, or tasks of daily living) and turn to nonfamily members only when familial sources of support are not available. Older adults, thereby particularly prefer the support of their spouses, if available, followed by their children and other relatives (Messeri et al., 1993). Cantor’s theoretical model provides a useful framework for studying EOL situations and decision-making. Indeed, adult children and spouses, and especially women, are commonly involved in the care of frail older adults (Pinquart & Sörensen, 2011). Furthermore, spouses are the most dedicated caregivers of all family members in terms of investment, endurance, and tolerance (Roberto & Blieszner, 2015). In addition, older adults usually prefer and expect family members to make medical decisions on their behalf in case of mental incapacity (High, 1994; Piers et al., 2013). However, Cantor’s theoretical model does not apply to everybody, and some individuals will deviate from this framework in certain circumstances to meet their needs and/or those of their loved ones (Carr & Khodyakov, 2007). For instance, married couples may ignore the highly normative choice of support from their spouse if they do not trust their partners (Boerner et al., 2013). Thus, although the family appears to be the preferred source of support among older adults, including for caregiving, the quality of family relationships also determines the choice of support persons.

The quality of family and social relationships contributes to the type of health behaviors individuals pursue. Several studies have documented the protective effects of high-quality social relationships on health behaviors (Cassel, 1976; Cobb, 1976). Compared to individuals with tenuous or strained family and friendship ties, individuals with close and supportive relationships are more likely to engage in health-enhancing behaviors (Mier et al., 2017; Peterson et al., 2019) and, consequently, to enjoy better health (House et al., 1988; Kiecolt-Glaser & Newton, 2001). According to Boerner et al. (2013), social support and social control are the two main explanatory mechanisms for these patterns. Social support allows individuals with meaningful social ties to become psychologically strengthened and encouraged by these relations. As a result, individuals benefitting from emotional and social support are more motivated to stay healthy for the good of those they care for and also experience improved mental and physical health due to this support (House et al., 1988; Thoits, 2011). By contrast, social control perspectives focus on the role of significant others in regulating individuals' health behaviors (Lewis & Rook, 1999). Significant others can directly monitor, encourage, remind, persuade or pressure a person to adopt or adhere to positive health behaviors (Thoits, 2011) that allow individuals to maintain and improve their health and well-being. Thus, support and control by close individuals, including family members, can encourage the adoption of health-enhancing behaviors.

Advance care planning (ACP) has been defined as a process that includes personal reflection and discussion with relatives and/or professionals about values and life goals as well as future care and treatment preferences, with the possible outcome of designating a healthcare proxy and making an advance directive (AD) (Rietjens et al., 2017; Sudore et al., 2017). ADs record patients’ treatment preferences and decisions for the possible event of future decision-making incapacity. ACP and ADs help to improve the EOL care experience by improving the quality of patient-physician communication and congruence in preferences between patients and their caregivers, as well as reducing decisional conflict, increasing preferences for comfort care, and encouraging the documentation of treatment preferences (Jimenez et al., 2018; Malhotra et al., 2022). Furthermore, the existence of ADs is associated with more out-of-hospital care, an increased focus on comfort care, and may contribute to reducing overtreatment at the end of life (Brinkman-Stoppelenburg et al., 2014). In the absence of ADs, an adult will be designated to make substitute medical decisions for the incapacitated patient. Appointed or default surrogates are often family members (Su et al., 2020), either through legal designation by the patient or the judicial system. Family members might experience surrogate medical decision-making as stressful and a burden (Peterson et al., 2018; Wendler & Rid, 2011), especially when they do not know the patient’s wishes (Marx et al., 2014). Therefore, discussing EOL preferences and completing ADs not only help maintain a good quality of life at the EOL but also reduce family’s stress and potential guilt in making an uninformed medical decision (Tilden et al., 2001). Despite this beneficial impact of ACP for the family, adults often approach such planning with hesitation, as thoughts of one’s imminent illness and ultimate death are potentially distressing and may trigger denial rather than active planning (Boerner et al., 2013; Carr, 2012b; Peterson et al., 2018). However, the availability of comforting and supportive relationships can mitigate distress (Thoits, 2011) and encourage positive health behaviors (Boerner et al., 2013), such as engagement in ACP.

In summary, families often play an important role at the end of life, and the quality of family relationships may impact how individuals engage in ACP. A measure of the quality of family relationships is the level of trust that individuals place in their family members. Indeed, trust is a key element of relationships and is included in most theoretical relational models (Wong & Sohal, 2002). Moreover, the literature generally sees trust as one of the three major dimensions of relationship quality, along with commitment and satisfaction (Athanasopoulou, 2009). Therefore, our study aims to evaluate the importance of older adults’ trust in their relatives regarding EOL issues for ACP. Specifically, we examine levels of trust adults aged 55 years have in their relatives for those who have discussed their EOL preferences, are aware of, approve and have completed ADs, and have designated a healthcare proxy in Switzerland. In addition, we investigate how trust in relatives regarding EOL issues is associated with these different approaches toward ACP. While previous studies have explored the importance of trust in the use of healthcare and patient-healthcare professionals’ relationships (Calnan & Rowe, 2007; Gilson, 2006; Ward, 2017; Yuan et al., 2007), as well as the role of relatives in influencing a person’s health behaviors (Lewis & Rook, 1999), there are—to the best of our knowledge—no previous studies that have investigated the relevance of the trust in relatives for engagement in ACP.

## Background

In Switzerland, ADs have been legally binding since 2013, when they were introduced into the Swiss Civil Code. Medical associations, patient organizations, and other private foundations have supplied templates for completing ADs. Typically, these form templates consist of sections that outline an individual’s treatment preferences, their stance on organ donation, and their chosen healthcare proxy. Some form templates also include questions about personal values, life’s meaning and quality, and associated fears and expectations. While individuals can complete these AD forms independently, it is generally recommended to do so under the guidance of trained professionals or healthcare specialists. Currently, Switzerland does not have a registry or centralized system to monitor the completion of ADs, often posing challenges in accessing them swiftly.

## Methods

Data come from a paper-and-pencil self-completion questionnaire about preferences, knowledge, attitudes, and behaviors towards EOL care planning (hereafter the EOL questionnaire) developed by the Swiss team of the Survey of Health, Ageing, and Retirement in Europe (SHARE) and palliative care experts of the Lausanne University Hospital. The EOL questionnaire was distributed at the end of the regular interview of SHARE in 2015 (wave 6) in Switzerland (Börsch-Supan, 2022). SHARE is a longitudinal, interdisciplinary, and cross-national data infrastructure that includes individual-level information on health, socio-economic status, social and family networks, and other life circumstances of older persons from 27 European countries and Israel. Switzerland has participated in SHARE since the beginning in 2004.

The Swiss SHARE sample was designed to be nationally representative of community-dwelling individuals aged 50 and older and their partners and was periodically refreshed to maintain its target population. Since the last SHARE Switzerland refreshment sample was drawn in 2011, we only include adults aged 55 and older in this research as a representative sample. A total of 2806 respondents participated in the SHARE regular interview in 2015 in Switzerland, and 94% of those also completed the EOL questionnaire, resulting in a sample of 2549 respondents. Retention of only observations with no missing data on all variables selected for the analysis results in a final analytical study sample of 1911 respondents.

### Measures

#### Outcome Variables

Our outcome variables come from the EOL questionnaire and translate the different stages involved in the ACP process.

EOL Preferences discussion: This variable is based on the question: “Some people communicate their preferences for the end of their life, while others do not. Have you ever had a discussion with someone about your wishes for the end of your life?” The answer categories were (1) “Yes” or (0) “No.”

AD awareness: The question related to this variable is: “Advance directives are a written statement in which an individual can describe his/her preferences for medical treatments and care in case he/she becomes incapable of making decisions. Individuals can also designate someone who can make medical decisions for them if necessary. This written statement is binding for medical providers and relatives. Prior to today, have you heard about advance directives?” Respondents answered by (1) “Yes” or (0) “No.”

Healthcare proxy designation: The question corresponding to this variable is: “Have you appointed someone in writing to make medical decisions for you should you not be able to make those decisions for yourself?” with response categories being (1) “Yes” or (0) “No.”

AD Completion: This variable is built on the question: “Have you completed a written statement about your wishes and refusals for medical treatments and care (advance directives)?” Respondents had to choose between (1) “Yes” and (0) “No.”

AD approval: This is a composite variable that captures respondents’ readiness and openness to formal communication of their preferences for EOL care and treatment. This variable includes respondents who reported having designed a healthcare proxy, or having completed ADs, or planning to complete ADs in the future. In the EOL questionnaire, if respondents had not completed an AD, they were asked if they planned to complete one in the future: “How likely is it for you to have a written statement about your wishes and refusals for medical treatments and care some day in the future?” The four response categories were grouped as follows in this study: “For sure” or “Very likely” coded as 1 and “Not very likely” or “Certainly not” coded as 0.

If applicable, respondents were also asked with whom they discussed their EOL preferences and ADs, and whom they designated as their healthcare proxy.

#### Independent Variable

Our key independent variable relates to the level of trust in relatives regarding EOL issues. Respondents were asked the following: “While some people fully trust certain persons or institutions, other people are apprehensive of them. With regard to end-of-life issues, to what degree do you trust relatives?” They were asked to rate their level of trust in relatives on a 4-point Likert scale ranging from “Completely” to “Not at all.” We dichotomized the four response categories into: “Somewhat,” “A little,” and “Not at all,” coded as 0 (subsequently referred to as “Absence of or limited trust in relatives”) and “Completely” coded as 1 (subsequently referred to as “Complete trust in relatives”).

#### Control Variables

We included sociodemographic characteristics of our respondents as control variables in our regression models. This information comes from the regular SHARE interviews: sex; age (reported in three groups: 55–64, 65–74, and 75 + years); education level (grouped in three categories based on the ISCED classification (UNESCO, 1997), that is, “low”: pre-elementary, elementary, and lower secondary education; “medium”: upper- and post-secondary education; and “high”: tertiary education); living with a partner in the same household (yes, no); having living children (yes, no); being able to make ends meet (easily, fairly easily, with difficulty); the type of place of residence (urban, rural area) and linguistic background as defined by the language used for the interview (German, French or Italian). Finally, respondents’ health was measured through self-reported limitations in basic and instrumental activities of daily living (ADLs and IADLs) (no limitation, 1+ limitation(s)) and self-rated health status (fair/poor, good/very good/excellent).

### Statistical Analysis

We first present weighted proportions for the characteristics of respondents included in the final analytical sample using calibrated cross-sectional weights available in SHARE (Börsch-Supan, 2022). Using multivariable logistic regression models, we explored the associations of EOL discussion, ADs awareness, approval and completion, and healthcare proxy designation with trust in relatives regarding EOL issues. Respondents’ sociodemographic and family characteristics, geographical location, and health status were included in the multivariable regression models as control variables. Average partial effects (APEs) were calculated to facilitate the interpretation of the logistic regression estimates. The estimated standard errors account for clustering at the household level to correct for potential unobserved dependencies between two observations coming from the same household. All data management and statistical analyses were conducted using Stata SE 17.0 (StataCorp LLC, College Station).

## Results

Table 1 presents the weighted respondents’ characteristics. Regarding ACP characteristics, 49.5% of older adults had ever discussed their preferences for their end of life with another person. 80.2% of respondents were aware of the existence of ADs before the introduction of the concept in the survey, and 79.6% of respondents approved of them, that is, they either had designated a healthcare proxy, completed an AD or were planning to complete one in the future. However, only a minority of respondents had already completed an AD (20.7%) or had designated a healthcare proxy in writing to make medical decisions for them should they lose decision-making capacity (16.4%). Concerning family relationship quality, 79.7% of respondents stated to have complete trust in their relatives regarding EOL issues.

**Table 1.** Distribution of Respondents’ Characteristics, Adults Aged 55+, SHARE Switzerland, 2015, *n* = 1911.

Variables	Weighted % (observations *n*)	95% confidence interval
Outcome variables		
End-of-life preferences discussion	49.5% (974)	[46.8–52.1]
Advance directive awareness	80.2% (1549)	[77.9–82.3]
Advance directive approval	79.6% (1532)	[77.3–81.6]
Advance directive completion	20.7% (415)	[18.7–22.9]
Healthcare proxy designation	16.4% (329)	[14.6–18.4]
Independent variable		
Complete trust in relatives regarding end-of-life issues	79.7% (1550)	[77.4–81.7]
Control variables		
Women	50.8% (1020)	[48.6–53.0]
Age groups		
55–64 years	49.9% (738)	[47.1–52.6]
65–74 years	28.9% (703)	[26.7–31.2]
75 + years	21.3% (470)	[19.3–23.3]
Education		
Low	16.4% (337)	[14.7–18.3]
Medium	66.3% (1247)	[63.8–68.7]
High	17.3% (327)	[15.4–19.4]
Partner living in household	72.4% (1499)	[69.9–74.8]
Having 1+ children alive	83% (1620)	[80.7–85]
Able to make ends meet		
Easily	56.1% (1106)	[53.2–58.9]
Fairly easily	30.7% (581)	[28.2–33.4]
With difficulty	13.2% (224)	[11.4–15.3]
Living in an urban area	46.9% (881)	[44.1–49.7]
Linguistic region		
German-speaking	74% (1417)	[71.4–76.4]
French-speaking	23.7% (443)	[21.4–26.2]
Italian-speaking	2.3% (51)	[1.7–3.2]
1+ limitations in activities of daily living	5.3% (101)	[4.3–6.6]
1+ limitations in instrumental activities of daily living	10.4% (202)	[9–12]
Fair/poor self–rated health	16.9% (322)	[15–18.9]

*Note*. weighted proportions and unweighted number of observations of the analytical sample.

**Figure 1.**
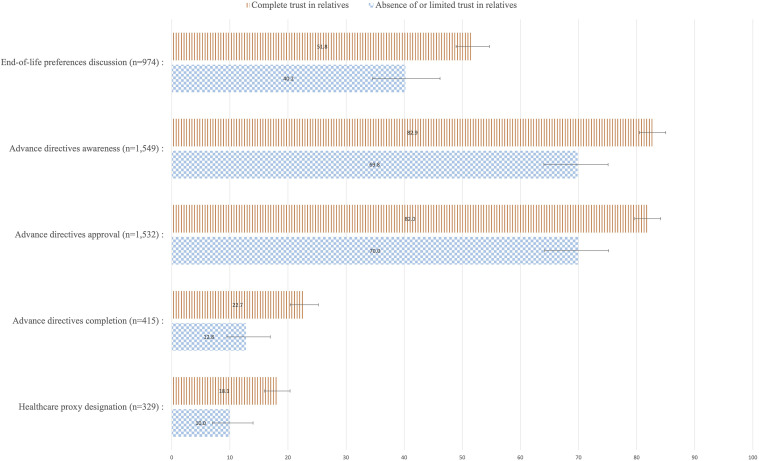
Respondents’ approaches towards Advance Care Planning by level of trust in relatives, weighted percentage, and 95% confidence intervals, adults aged 55+, SHARE Switzerland, 2015.

Figure 1 displays the weighted percentage of respondents’ ACP outcomes by level of trust in their relatives, along with 95% confidence intervals. Proportions of respondents who discussed their EOL preferences; were aware of the existence of ADs; approved ADs; completed ADs, and designated a healthcare proxy were systematically and significantly higher among respondents with complete trust in relatives regarding EOL issues than among those with limited or absence of trust in their relatives.

The first column of Table 2 presents estimates of the partial associations of sociodemographic, family, geographical, and health characteristics with complete trust in relatives regarding EOL issues. Compared to men, women were four percentage points more likely to display complete trust in their relatives regarding EOL issues (APE: .04, *p* < .05). Complete trust in relatives was also positively associated with a high level of education (APE: .10, *p* < .001), living with a partner in the same household (APE: .06, *p* < .05), and having living children (APE: .08, *p* < .01). Compared to German-speaking respondents, respondents with a French- or Italian-speaking background were less likely to display full trust in their relatives regarding EOL issues (APE: −.10, *p* < .001 and −.13, *p* < .05, respectively). Respondents with at least one limitation in basic activities of daily living also had lower levels of trust in relatives with regard to EOL issues (APE: −.11, *p* < .05).

**Table 2.** Average Partial Effects (APEs) Based on Logistic Regressions of Approaches Toward Advance Care Planning on Complete Trust in Relatives, Adults Aged 55+, SHARE Switzerland, 2015, *n* = 1911.

	Complete Trust in Relatives	End–of–life Preferences Discussion	Advance Directive Awareness	Advance Directive Approval	Advance Directive Completion	Healthcare Proxy Designation
Level of trust in relatives regarding end–of–life issues (ref.: Absence of or limited trust)						
Complete trust		.08^**^ (.03)	.05^*^ (.02)	.06^*^ (.02)	.07^**^ (.02)	.07^***^ (.02)
Gender (male)						
Female	.04^*^ (.02)	.16^***^ (.02)	.05^**^ (.01)	.11^***^ (.02)	.08^***^ (.02)	.04^**^ (.02)
Age group (55–64 years)						
65–74	.02 (.02)	.01 (.03)	−.00 (.02)	.00 (.02)	.07^***^ (.02)	.07^***^ (.02)
75+	.02 (.02)	−.01 (.03)	−.05^*^ (.02)	−.07^**^ (.02)	.16^***^ (.03)	.11^***^ (.03)
Education (low)						
Medium	.02 (.03)	.07^*^ (.03)	.06^**^ (.02)	.12^***^ (.03)	.00 (.03)	−.00 (.02)
High	.10^***^ (.03)	.08 (.04)	.07^**^ (.03)	.17^***^ (.03)	.04 (.04)	.02 (.03)
Partner living in household (No partner)						
Partner	.06^*^ (.02)	.07^*^ (.03)	.03 (.02)	.05^*^ (.02)	−.04 (.03)	−.04 (.02)
Number of children alive (No child)						
1+ child	.08^**^ (.03)	.04 (.03)	−.02 (.02)	−.03 (.02)	−.04 (.03)	−.05 (.03)
Make ends meet (easily)						
Fairly easily	−.01 (.02)	−.02 (.03)	−.01 (.02)	−.04^*^ (.02)	−.01 (.02)	−.02 (.02)
With difficulty	−.03 (.03)	−.02 (.04)	−.05 (.03)	−.04^*^ (.03)	−.03 (.03)	−.01 (.03)
Residence area (rural) Urban area	−.00 (.02)	.03 (.02)	−.01 (.02)	−.00 (.02)	−.01 (.02)	−.01 (.02)
Linguistic region (German–speaking)						
French-speaking	−.10^***^(.02)	−.14^***^ (.03)	−.45^***^ (.03)	−.24^***^ (.03)	−.17^***^ (.02)	−.12^***^ (.02)
Italian-speaking	−.13^*^ (.06)	−.16^*^ (.07)	−.34^***^ (.07)	−.31^***^ (.07)	−.10 (.06)	−.06 (.06)
Limitations in activities of daily living (ADL) (No limitation)						
1+ ADL limitations	−.11^*^ (.05)	.10 (.06)	.02 (.03)	.08^*^ (.03)	.08 (.05)	.04 (.05)
Limitations in instrumental activities of daily living (IADL) (No limitation)						
1+ IADL limitations	.00 (.03)	.04(.04)	.00(.03)	−.00 (.03)	.01 (.03)	.10^*^ (.04)
Self–rated health (excellent/very good)						
Fair/poor	−.05 (.03)	−.01 (.03)	−.05^*^ (.02)	−.03 (.03)	−.01 (.03)	−.06^**^ (.02)
Observations	1911	1911	1911	1911	1911	1911

*Note*. APEs and standard errors in parentheses have the following significance levels *p < .05, **p < .01, ***p < .001. Concerning the interpretation of APEs, for instance, the number on the top–left means that compared to men, women are four percentage points more likely to display a complete trust in their relatives regarding end–of–life issues.

Columns two to six of Table 2 display estimated APEs based on multivariable logistic regression models for having had a discussion of EOL preferences, AD awareness, AD approval, and AD completion, as well as healthcare proxy designation on complete trust in relatives regarding EOL issues, controlling for sociodemographic and family characteristics, geographical location, and health status. Complete trust in relatives with regard to EOL issues was positively associated with all five outcomes: having had a discussion of EOL preferences (APE: .08, *p* < .01), AD awareness (APE: .05, *p* < .05), AD approval (APE: .06, *p* < .05), AD completion (APE: .07, *p* < .01), and healthcare proxy designation (APE: .07, *p* < .001).

**Figure 2.**
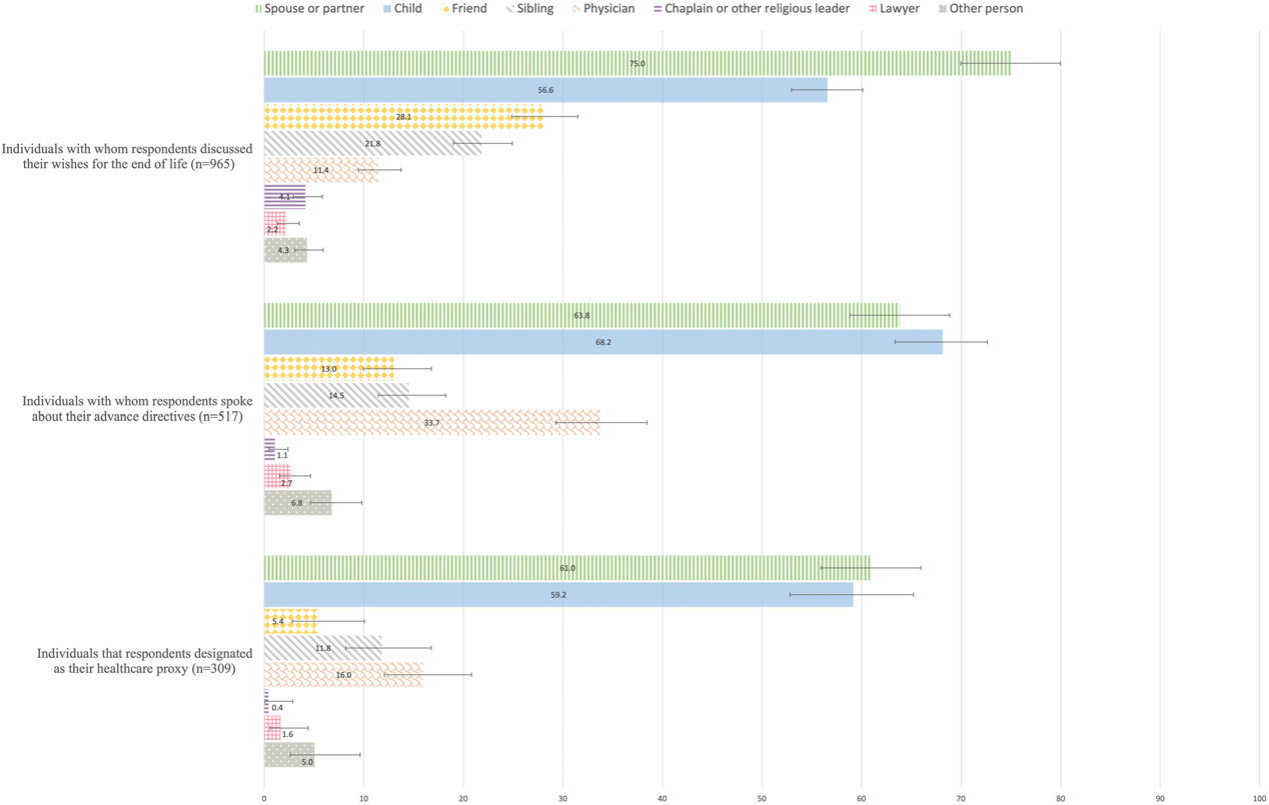
Respondents’ interlocutors for Advance Care Planning, weighted percentages, and 95% confidence intervals, adults aged 55+, SHARE Switzerland, 2015 **(***Note*. Respondents could choose more than one category).

Figure 2 shows the weighted proportions of types of individuals with whom respondents discussed their wishes for the end of life, spoke about their ADs, and who they have designated as their healthcare proxy, along with 95% confidence intervals. Respondents mainly selected their spouses and children as partners for their discussions about their EOL preferences and their ADs, and as designated healthcare proxies.

## Discussion

Our study provides new insights into the relationship between important ACP outcomes and trust in relatives regarding EOL issues among older adults in Switzerland. We found that complete trust in relatives regarding EOL issues is positively associated with having discussed EOL one’s preferences, being aware of, approving and completing ADs, and having designated a healthcare proxy. These findings indicate that the quality of family relationships is important for how older adults view and engage in ACP. The following paragraphs further discuss the likely implication of trust in relatives regarding EOL issues for how individuals comprehend and engage in ACP. The associations of control variables with ACP characteristics are not discussed as they are comparable to those found and described in a previous study based on the same dataset (Vilpert et al., 2018).

### End-of-Life Preferences Discussion

Our study revealed that although most respondents completely trusted their relatives with regard to EOL issues (79.7%), only about half of respondents (49.5%) have already had a discussion of their EOL preferences with another person. Our results are consistent with other studies, which show that most older adults tend not to discuss preferences for their long-term care, even with trusted others (Garavan et al., 2009; Hopp, 2000; Pautex et al., 2018). There are many barriers to discussing ACP, both on the side of the person affected by the potential care plans as well as on the side of other potentially involved persons. The prospect of one’s death is one of the main obstacles to these discussions (Winland-Brown, 1998). Death is a difficult and often emotionally charged topic to address, which may trigger feelings like anxiety, fear, discomfort, sadness, and grief (Peterson et al., 2018). Additionally, EOL discussions are often avoided because of fears of the potential embarrassment it may create for others (Greene & Adelman, 1996). Family members and significant others are not always open to EOL discussions. Emotionally concerned and affected by the future prospects of their loved one, close relatives may complicate or avoid EOL conversations, especially when they are unable to accept the advanced nature of a loved one’s disease or her preferences concerning EOL care (Larson & Tobin, 2000). Another frequent barrier to EOL discussions is a previous breakdown of family relationships or a lack of close family altogether (Peterson et al., 2018; Sharp et al., 2013). Consistent with this statement, our results show that the proportion of individuals who have discussed their EOL preferences is higher among respondents who trust their family completely than among those who do not.

Our findings showed that when a discussion about EOL preferences took place, it usually involved the respondents’ partners and/or children. As such, the family appears to be a privileged partner for sensitive discussions about older adults’ EOL preferences. Since discussing EOL preferences is often highly emotional, unsettling and requires a trusting environment (Peterson et al., 2018), not everybody represents a suitable discussion partner for such conversations (Foster, 2014). Some individuals prefer to talk about their end of life with professionals and persons who are not personally affected by the matter, such as healthcare providers. In contrast, other individuals prefer to engage in EOL discussions with more intimate communication partners, such as close relatives who have known them for some time and who tend to be more readily available.

An additional element that may encourage individuals to discuss EOL preferences with a relative is the belief that they will be better listened to and understood by their relatives. Indeed, EOL discussions appear to be sensitive to the functioning of families (e.g., sharing thoughts and feelings, collaborative problem solving) and can be facilitated when family relationships are marked by open communication and sharing of each other’s fears and feelings (Boerner et al., 2013), which generally requires a high level of trust. In our study, trust in relatives regarding EOL issues can be viewed as a proxy measure of the quality of family relationships. We found that complete trust in relatives was positively associated with having had a discussion of one’s EOL preferences keeping other characteristics fixed. Similarly, Boerner et al. (2013) found that increased family functioning raises the odds of having EOL-related discussions. These findings suggest that the end of life and death are sensitive subjects that are more easily addressed in a context of trust that seems to be often found within families.

### Advance Directives Awareness

Our data further showed that while most respondents were aware of the existence of ADs, rates of awareness were even higher among persons who stated to completely trust their relatives with regard to EOL issues (82.9%) than among those who did not (69.8%). This finding was also robust to controlling for a comprehensive set of individual characteristics. This result suggests that a trusted family may be involved in raising awareness of ADs among older adults. Indeed, it has been shown that many individuals learn about ADs from family, friends, personal attorneys, and media, while fewer individuals learn about it from healthcare providers (Duke et al., 2013). In our study, the potential role of the family in AD awareness is further supported by the close link between AD awareness and EOL discussions. Specifically, respondents who have had a discussion about their EOL preferences were also more likely to have heard about ADs (APE: .14, *p* < .001, results not shown). Since most respondents discussed their EOL preferences with their close relatives (partner, children, sibling), ADs may have been a topic raised by their discussion partners, who may, therefore, have contributed to increasing AD awareness among respondents.

### Advance Directives Approval and Completion

We found that complete trust in relatives is associated with higher approval and completion rates for ADs. These results are consistent with a positive impact of social support and control on health behaviors. Social relationships can affect health, either by promoting a sense of meaning or coherence that enhances health (Antonovsky, 1979) or by fostering health-promoting behaviors such as proper sleep, exercise, adherence to medical regimens, or getting needed medical care (Umberson, 1987). Hence, we might expect that persons with high-quality relationships will be more motivated to engage in ADs because their close relatives encourage them to do so (Umberson et al., 2010) and because they know that it may help to protect their loved ones from difficult decisions regarding EOL care as a potential future healthcare proxy (Carr, 2012a). Previous studies have shown that individuals with supportive and close relationships are more likely than those with strained or tenuous connections to engage in health-enhancing behaviors and enjoy better health (House et al., 1988; Kiecolt-Glaser & Newton, 2001). Additionally, Boerner et al. (2013) showed that better overall family functioning and more frequent emotional support from a spouse and/or child increased the odds of engaging in ACP. They found that with each one-point increase (on a four-point scale) in family functioning, the odds of engaging in both formal (i.e., living will or durable power of attorney for health care) and informal (i.e., discussions about preferences) EOL preparations nearly doubled. Our results corroborate this finding by showing that complete trust in relatives is associated with increased support for and completion of ADs.

Although the positive association between complete trust in relatives and AD completion is coherent with the health-enhancing impact of social support and control, we may have expected the opposite result: respondents with more trust in their family may be less likely to have ADs. Indeed, consistent with the hierarchical compensatory model (Cantor, 1979), married couples might prefer to sidestep formal ADs because they believe their spouse knows their medical care preferences and will make appropriate medical decisions on their behalf if required (Kelly et al., 2012). In this context, one would expect that complete trust in relatives decreases the chances of having ADs, as found by Piers et al., (2013). Absence or a limited trust in one’s relatives could, therefore, increase the use of ADs, as patients may want full control over their EOL decisions, but this is not what our data indicate.

Many individuals complete ADs as they do not want to be a burden on their family (Douglas & Brown, 2002; Duke et al., 2013; Libbus & Russell, 1995). Individuals who fully trust and care about their loved ones may want to protect them from the pain of having to make difficult decisions about stopping or prolonging treatment and from potential family conflicts if there is family disagreement about the appropriate decision (Carr & Khodyakov, 2007). ADs help prevent family guilt over treatment decisions (Douglas & Brown, 2002) and reduce family stress associated with honoring patient wishes in the EOL decision-making (Duke et al., 2013; Tilden et al., 2001; Wilson, 2000). Hence, the completion of ADs may reflect a desire to facilitate decision-making for the family in case of incapacity (Douglas & Brown, 2002) and a wish not to leave relatives alone during possibly ethically and morally challenging decision-making situations at the EOL (Pautex et al., 2018). In line with this argument, we show that if respondents have completed ADs, they mainly discuss their ADs with their partners and/or children. By contrast, the motivation to spare one’s family from complicated medical decision-making at the EOL may be lower in the context of strained family relations (Boerner et al., 2013).

### Designation of a Healthcare Proxy

In our data, the share of respondents who designated a healthcare proxy was higher among those who completely trusted their relatives regarding EOL issues than among those who did not. In addition, most designated healthcare proxies were close relatives (partners and/or children). We also found a positive partial association between complete trust in one’s relatives and the designation of a healthcare proxy adjusting for other characteristics. Consistent with other studies (Boerner et al., 2013; Carr & Khodyakov, 2007), older adults who feel that they can trust family members are more likely to appoint them as healthcare proxies than those who do not trust their relatives. Hence, appointing a family member as a healthcare proxy appears to be contingent on trust in relatives.

Similarly, and consistent with the hierarchical compensatory model (Cantor, 1979), the findings of Hopp (2000), Carr and Khodyakov (2007) and Luck et al. (2017) suggest that older adults mainly select spouses and members of the younger generation as potential surrogate decision-makers and turn to other relatives and nonrelatives only when these close family members are not available. Boerner et al. (2013) show that as the frequency of spousal criticism increases, the odds of naming one’s spouse as a durable power of attorney for healthcare decreases. Individuals seem to name the person that is perceived to best listen to them and to best represent their views in the EOL decision-making process as their healthcare proxy. Critical spouses may be perceived as not fully trustworthy for surrogate medical decision-making because they have difficulty empathizing and advocating for their partner’s wishes. Hence, Boerner et al. (2013) suggest that married couples may bypass the highly normative choice of their spouse as the designated healthcare proxy when the relationship is problematic. The absence of or limited trust in relatives regarding EOL issues may indicate poor family relationships that could discourage the appointment of a healthcare proxy, which would usually be a family member.

Overall, our findings show a consistent relationship between trust in relatives and the different aspects of ACP—discussion, AD awareness, approval and completion, and healthcare proxy designation—which are important to consider separately. Each aspect represents a distinct stage in the ACP process, and though related, the same individuals may show different attitudes to these stages. Our results, therefore, contribute to the literature by providing a nuanced understanding of how trust is associated with ACP at different stages. From a practical perspective, detailed observation of the ACP process and its association with trust in relatives provides important information for designing tailored interventions aimed at increasing individuals’ engagements in ACP.

### Socio-Cultural Patterns of Trust and ACP Characteristics

The proportion of respondents who declared to have complete trust in relatives regarding EOL issues is high in our study, reflecting the high level of trust that individuals living in Switzerland place in Swiss public institutions compared to other OECD countries (OECD, 2021). In addition, some sociodemographic and cultural/regional characteristics are associated with complete trust in relatives. We observe that population groups who tend to have less trust in their relatives regarding EOL issues, such as men, individuals with low education levels, and individuals living in French- and Italian-speaking regions, also tend to have less favorable approaches toward ACP. We will attempt to provide potential explanations for these socio-cultural patterns.

Women usually care more about building and maintaining relationships than men (Haselhuhn et al., 2015) and are more likely to be involved in family care (Wright et al., 2008). They usually express a greater sense of responsibility toward family members and devote more time to caregiving (Aronson, 1992). Women are also more open to engaging in all topics of death and dying (Seifart et al., 2020) and are less likely to deny life’s problems (Verbrugge, 1985). Additionally, in similar assessments of illness and injury, women are more likely than men to seek care or assistance (Verbrugge, 1985), which might explain their higher engagement in ACP.

Similarly, regarding education level, a successful professional experience is likely to make individuals more prone to trusting others (Alesina & La Ferrara, 2002). Schooling provides an environment with opportunities for socialization that can engender positive attitudes toward people in general (Zanin, 2017). Schooling not only helps improve communication and social skills (Glaeser et al., 2000) but also helps increase understanding and awareness of more scientific topics. Moreover, newspapers and magazines are more likely to be used regularly by people with higher levels of education, and more educated people are more likely to read about health than less educated people (Wade & Schramm, 1969). Following this, more educated people may be more aware and willing to engage in ACP and have less difficulty communicating the EOL care they would like to receive, compared to less educated people (Desharnais et al., 2007; Peterson et al., 2019).

Finally, regarding cultural differences and similarities, Egli et al., (2017) argue that bonds between parents and children are much looser in German culture than in French or Italian culture. In addition, German-speaking adults value more independent family patterns. For instance, German parents tend to offer more choices and fewer constraints on children than French parents (Albert et al., 2011). As more independence usually requires a higher level of trust, this prevalence of independence among German-speaking families may likely be related to higher trust. It might also affect individuals’ willingness to engage in ACP, as we have seen that ADs can be used to preserve autonomy.

More complex mechanisms might link individual characteristics, trust in relatives, and approaches toward ACP that need to be investigated in further research using more detailed measurements.

## Limitations

Our study has several limitations. A first limitation relates to missing responses. Older adults, individuals with low education levels, and those in poor health were slightly more likely not to have answered the outcome questions. However, non-response rates in these groups remained, on average, below 10%. It thus seems unlikely that sample selection related to missing responses is driving our results. Another study limitation is that some respondents may declare not to trust their relatives because they simply don’t have relatives. However, as our models adjusted for family structure (being partnered, having living children), family composition is unlikely to play a major role in the association between trust in relatives and approaches toward ACP. A third limitation concerns our assumption that trust in relatives is used as a proxy variable of the quality of family relationships. While a low level of trust is often a reflection of a problematic relationship, it is possible for two individuals of the same family to trust each other completely without having a close relationship, which would overestimate the importance of good family relationships in engaging in ACP. A fourth limitation is that we did not directly measure the role of relatives in respondents' ACP behaviors. While trust in relatives was positively associated with engagement in the ACP process, this does not necessarily imply that the family members actively moderated potential stress associated with ACP or directly encouraged older adults to engage in ACP.

## Conclusion

Trust in relatives regarding EOL issues correlates in how older adults plan their EOL care. A higher level of trust in relatives regarding EOL issues is linked to a more positive attitude and more proactive behavior toward ACP. Our findings highlight how the family relationships—as measured through trust in relatives—is related to ACP processes.

Previous research has shown that close and trusting relationships usually increase the likelihood of engaging in health-enhancing behaviors. We show that this relationship also holds true for proactive ACP. Hence, the family continues to be important at the end of life and for EOL care, even in more individualistic societies. While tools such as ADs aim to guarantee individuals’ autonomy and self-determination in all circumstances, the awareness, approval, and use of ADs seem to remain strongly linked to individuals’ family contexts. Therefore, healthcare providers involved in ACP should offer—with the patient’s consent—to include family members in the different stages of the process. Finally, population-wide information and education on EOL situations is crucial, given the substantial likelihood that individuals will find themselves involved as patients or family members navigating EOL issues and decisions.
